# Prevalence, determinants of chronic rhinosinusitis and its impact on quality of life among students in Karachi, Pakistan

**DOI:** 10.2144/fsoa-2022-0050

**Published:** 2023-01-10

**Authors:** Tafazzul Hyder Zaidi, Mubashir Zafar, Zafar Haleem Baloch, Alisha Shakeel, Nouman Mansoor Ali, Beenish Nisar Ahmed, Muhammad Amash Khan, Rafia Masood, Iman Fatima, Sarah Shakeel

**Affiliations:** 1 Department of Community Medicine, Jinnah Sindh Medical University, Karachi, 75510, Pakistan; 2 Department of Family & Community Medicine, College of Medicine, University of Hail, Hail, 2440, Kingdom of Saudi Arabia; 3, Anatomy Department, Sind Medical College, Jinnah Sindh Medical University, Karachi,75510, Pakistan; 4 Medical students, Sindh Medical College, Jinnah Sind Medical University, Karachi, 75510, Pakistan; 5 Otolaryngology Department, Khan Research Laboratory (KRL) Hospital, Islamabad, 44000, Pakistan; 6 University of Karachi, Karachi, 75270, Pakistan

**Keywords:** chronic rhinosinusitis, quality of life, smokers, students

## Abstract

**Background:**

Chronic rhinosinusitis (CRS) is a common public health issue among students.

**Methodology:**

A total of 300 undergraduate students were selected through multistage cluster sampling from three public-sector universities. Sino-nasal Outcome Test (SNOT-22) and Rhinosinusitis Disability Index (RSDI) were used for determining the quality of life. Chi-square and independent *t*-test were used.

**Results:**

About 46% and 54% (p = 0.001) of social science and health science students were suffering from CRS, respectively. Around 7% and 9% of CRS patients had poor quality of life according to SNOT-22 and RSDI, respectively (p = 0.042 and p = 0.032, respectively).

**Conclusion:**

Quality of life was affected in all domains of SNOT-22 and RSDI.

## Plain language summary

Rhinosinusitis is a common problem in society. Students are affected commonly because of exposure and low awareness. This study determines the burden of flu among medical and nonmedical students. We conducted the survey in public-sector universities. Results of study found that medical students were more suffering from chronic flu. This flu affected the social and family life. Common symptoms are pain in the face, pain in the eye and continuous headache. There is need to increase awareness among students regarding flu.

The upper respiratory tract is the first line of protection from air pollution. Common air pollutants are tobacco, traffic and particulate matter 2.5 [1]. Traffic-related air products (TRAPs) are the emissions of harmful gaseous and pollutant mixtures into the air which originate from combustible and noncombustible sources directly or indirectly [2]. TRAPs along with tobacco smoke precipitate respiratory diseases, especially sinusitis. According to the American Academy of Pediatrics, rhinosinusitis is inflammation of “nose and paranasal sinuses”. Based on causative organism and time duration, sinusitis is further classified into acute, subacute and chronic sinusitis [3].

Tobacco smoke is an environmental hazard that can impact the respiratory tract through first and secondhand smoke. The prevalence of chronic rhinosinusitis (CRS) is closely associated with active and passive cigarette smoke [4]. A systematic review was done to determine the relationship between sinusitis and secondhand smoke exposure, and results found a positive correlation between them [5,6]. It was learned that cessation of smoking is correlated with improvements in sinonasal symptoms [7].

There has been a substantial rise in respiratory problems in developing countries due to an increase in exposure to smoking. A previous study's results found that only a few hours spent every day on foot along busy roads will a give a significant rise in respiratory symptoms [8]. Exposure to indoor and outdoor air pollution is a major risk to respiratory health worldwide particularly in low- and middle-income countries [9]. A study examining the relationship between CRS and outdoor air pollution in Germany showed the consistent statistical impact of pollution on CRS prevalence [10]. Improvements in air quality are related to lower incidence of hay fever and sinusitis [11]. Owing to proximity to vehicular emissions, one study's results found severe respiratory illness in traffic officers in an Indian district [12]. There are benefits to wearing masks as a preventive measure against air pollution during traffic control by traffic wardens. One study showed that traffic police who did not use protective masks had higher relative risks of respiratory diseases [13]. A study which was conducted in secondary schools found that those students who had CRS experienced poor quality of life (QoL) [14], and CRS increased morbidity among these students [15]. The objective of this study is to determine the prevalence, determinants and impact of chronic sinusitis on the health-related QoL among students.

## Materials & methods

### Operational definition

CRS is defined as nose and paranasal sinus inflammation and it features two symptoms, which are mucopurulent secretion and obstruction of nasal pathway, and associated symptoms of facial pain and decreased sensation of smell for 4 months.

### Study setting, sampling technique & study design

This cross-sectional study was carried out on 300 undergraduate students from three public-sector universities. Study participants were selected from the health science and social science college through multistage cluster sampling. The study period was from February to April 2022.

### Sample size

For determining the sample size, the WHO sample size calculator was used: this is the validated software for calculating the sample for health studies (Lemeshow, S. *et al.*, Adequacy of sample size in health studies [Chichester, John Wiley, 1990; published on behalf of the WHO]). The parameters for sample size calculation are margin of error 5%, 95% CI and 27% prevalence from the previous study [16]. The minimum sample size required for this study was 300. Total sample for this study was 300.

### Study variables & inclusion & exclusion criteria

The dependent variables were the status of patient being chronic sinusitis positive or negative and QoL score. Independent variables were age, gender, student's area of study, smoking status, physical, functional and emotional domain of Sino-nasal Outcomes Test (SNOT-22) index, and Rhinosinusitis Disability Index (RSDI). CRS was diagnosed through self-reported physician diagnosis. Those students giving consent to participate in the study were included in the study, and those students with a chronic respiratory problem other than CRS were excluded from the study.

### Data collection procedure

Written informed consent was taken and data was collected by distributing structured questionnaires to the study participants. In order to standardize the questionnaires, a pilot study was conducted among participants to check the content validity. The questionnaire was divided into three sections: the first section based on demographic data, the second section based on symptomatology of QoL on the basis of SNOT-22 criteria and the third section containing RSDI components [16].

SNOT-22 is the most commonly used test for determining the QoL among sinusitis patients [17]. This test comprises 22 questions and is divided into four sections: first, nasal; second, otologic; third, sleep; and fourth, emotional. The nasal section includes questions regarding symptoms of sneezing, running nose, discharge, smell and cough. The otologic section includes questions regarding vertigo and dizziness. The sleep sections include questions regarding sleeping, walking in night tired, fatigue and decreased productivity. The emotional domain includes questions regarding frustration and sadness [18]. The results are categorized as mild (1–20), moderate (>20–50) and severe (>50) [15]. RSDI consists of 30 questions. It consists of four sections: physical (11 questions), functional (9 questions) and emotional (10 questions). The validity and reliability of RSDI are 0.97 and 0.87, respectively [7]. It is evaluated by Likert scale, which is five points (0 = never, 1 = almost never, 2 = sometimes, 3 = almost always, 4 = always), and scores summed to a maximum of 120 and minimum score of 0 [19]. Sinusitis variables were created and the response calculated through point scores from SNOT-22 scales. There were three categories of response: ‘never’ and ‘almost never’ were considered normal, ‘sometimes’ was considered mild, ‘almost always’ was considered moderate and ‘always’ was considered severe sinusitis. A higher score reflected poor health-related QoL.

### Statistical analysis

Data was analyzed using SPSS software version 26. Descriptive statistics are presented as mean scores, standard deviation or medians. Histogram and Kolmogorov–Smirnov test was used to determine the normal distribution of data. Inferential statistical analysis (Chi-square test for qualitative variable and *t*-test for quantitative variable) was used to test the relationship between SNOT-22 scores and RSDI scores with demographic characteristics. A p-value of <0.05 was considered statistically significant.

### Ethical consideration

The study protocol was approved from the ethical review board of Jinnah Sindh Medical University and informed consent was obtained for each participant before data collection. A written informed consent form was attached with each questionnaire. Participants first read and signed informed consent, and then data collection started.

## Results

The mean age of the participants was 23 years ± 0.53 standard deviation. The majority (57%) of the participants were in the age group of 18–21 years, most (59.7%) were female participants, only 10.7% were smokers, and total mean scores of SNOT-22 and RSDI were 69.23 (5.8 standard deviation) and 45.40 (12.3), respectively. A total of 49.7% of participants had CRS (Table 1).

**Table 1.** Sociodemographic characteristics of study participants (n = 300).

Characteristics	Frequency (n)	Proportion (%)
Age (years) mean ± SD 18–21 22–27	23 ± 0.530 171 129	57 43
Sex Male Female	121 179	40.3 59.7
Students' area of study Medical Nonmedical	150 150	50 50
Smoking Ever smoker Never smokes	32 268	10.7 89.3
Sinonasal Outcome Test score Nasal domain Otologic domain Sleep domain Emotional domain Total score	Mean (SD) 14.7 (2.3) 7.9 (1.5) 29.5 (3.9) 6.3 (1.2) 69.2 (5.8)	Range 0–35 0–15 0–50 0–10 0–110
Rhinosinusitis Disability Index score Physical domain Functional domain Emotional domain Total score	Mean (SD) 2.21 (0.65) 1.36 (0.21) 1.49 (0.33) 45.40 (12.3)	Range 0–11 0–7 0–6 0–72

SD: Standard deviation.

Common symptoms affecting the QoL were eye pain which makes it difficult to read (28.3%), pain in the face which makes it difficult to concentrate (25.3%) and sniffing which irritates friends and family (23.3%) (Table 2).

**Table 2.** Symptoms that affect the quality of life of study participants.

Symptoms	Frequency (n)	Proportion (%)
The pain or pressure in my face makes it difficult for me to concentrate	76	25.3
The pain in my eyes makes it difficult for me to read	85	28.3
I have difficulty stooping over to lift objects because of face pressure	46	15.3
I have difficulty with strenuous yard work and housework	65	21.7
Straining increases or worsens my problem	64	21.3
I am inconvenienced by my chronic runny nose	67	22.3
Food does not taste good because of my change in smell	54	18
My frequent sniffing is irritating to my friends and family	70	23.3
I don't sleep well	67	22.3
I have difficulty with exertion due to my nasal obstruction	59	19.7
My sexual activity is affected	11	3.7
I feel restricted in performance of my routine daily activities	81	27
I avoid traveling	49	16.3
I miss work or social activities	56	18.7

QoL scores (SNOT-22 and RSDI) were significantly associated with the study area and smoking status of students (Table 3).

**Table 3.** Health-related quality score (Sino-nasal Outcome Test) and Rhinosinusitis Disability Index associated with sociodemographic characteristics among study participants (n = 300).

Characteristics	Frequency (n)	SNOT-22 Mean (95% CI)	RSDI Mean (95% CI)	p-value
Age, years 18–21 22–27	171 129	14.70 (11.97–16.41) 15.94 (14.22–19.77)	5.14 (4.23–6.05) 4.94 (4.04–5.84)	0.116
Sex Male Female	121 179	14.77 (12.28–17.27) 15.82 (13.43–18.21)	5.34 (3.28–5.74) 5.90 (4.37–6.11)	0.560
Students area of study Medical Nonmedical	150 150	15.85 (13.34–18.35) 14.95 (12.52–17.38)	5.40 (4.54–6.26) 4.71 (3.74–5.68)	0.011
Smoking Current smoker Never smokes	32 268	15.78 (13.87–17.68) 12.70 (8.78–16.62)	6.63 (4.25–5.58) 4.91 (3.85–8.64)	0.042

**Figure 1.**
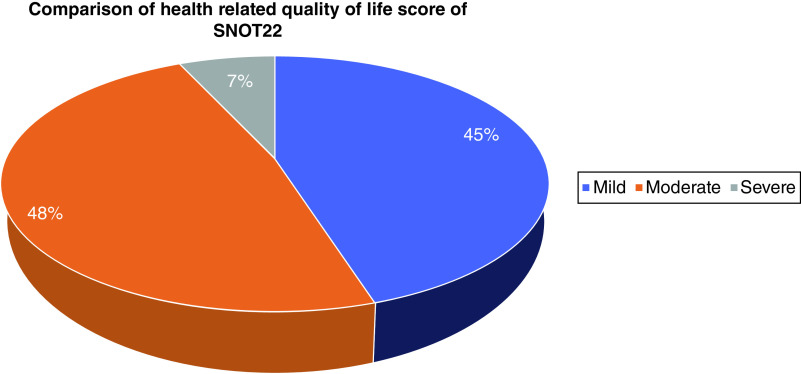
Comparison of health-related quality of life score based on Sino-nasal Outcome Test (p = 0.043).

Participants' QoL affected based on SNOT-22 showed a mild 45%, moderate 48% and severe 7% effect on QoL. This difference is statistically significant (p = 0.004) (Figure 1).

**Figure 2.**
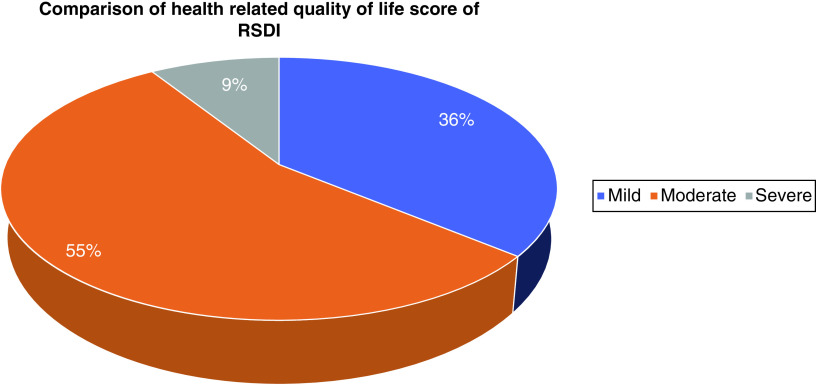
Comparison of health-related quality of life score based on Rhinosinusitis Disability Index (p = 0.032).

Participants' QoL affected based on RSDI showed a mild 36%, moderate 55% and severe 9% effect on QoL. This difference is statistically significant (p = 0.004) (Figure 2).

**Figure 3.**
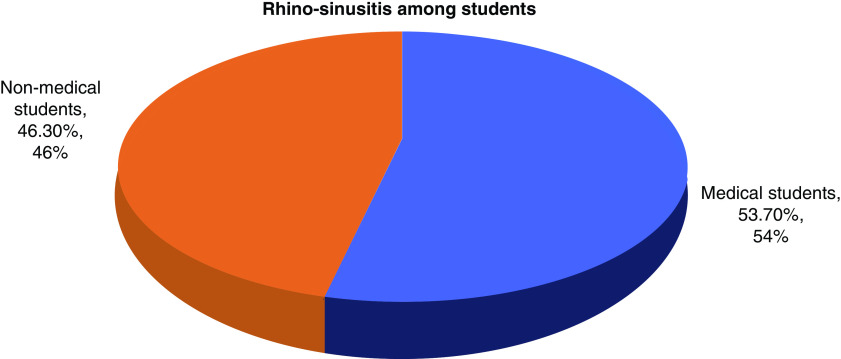
Rhinosinusitis among medical and nonmedical students (p = 0.00).

Medical students showed a greater prevalence of rhinosinusitis compared with nonmedical students. This difference is statistically significant (p = 0.000) (Figure 3).

## Discussion

This is the first study to determine CRS prevalence and its association with QoL among students in Pakistan. The majority of students had high scores for SNOT-22 and RSDI which indicate poor QoL.

The study found that the mean score of total SNOT-22 was 69.2, and mean scores of the different SNOT-22 domains were 14.7, 7.9, 29.5 and 6.3 for nasal, otologic, sleep and emotional domains, respectively. The mean score of total RSDI was 45.40, and mean scores of the different RSDI domains were 2.21, 1.36 and 1.49 for physical, emotional and functional domains, respectively. This result is consistent with other study results [20,21]. The reason for this result is exposure to outdoor pollution. When the SNOT-22 and RSDI total score was compared with age groups, higher scores were reported in the age group 22–27 years. This result is similar to studies which were done among CRS students [22–25]. This result is due to thinning of the lining of the epithelium among this age group, which brings more complications for the sinus and more frequently affected sinuses.

This study found that students' commonly experienced symptoms of QoL were pain in the face (25.3%), pain in the eyes (28.3%), difficulty working due to runny nose (22.3%) and difficulty in sleeping (22.3%). These results are consistent with other study results [26]. These results are due to the runny nose and chronic sore throat which affect the QoL, because these symptoms lead to headache, low focus and feeling unhealthy [26]. The study found that having facial pain or pressure makes it difficult for students to concentrate and perform well. This was supported by a study on cystic fibrosis which showed facial pain was significant in affecting in QoL due to physical activity limitation [27].

Participants with higher SNOT-22 scores reported worse emotional outcomes, similar to a study which showed that symptoms in CRS patients frequently made them vulnerable to depression [28]. Participants' RSDI scores were also high, and this result is consistent with other study results which found that participants with rhinologic disease have lower physical scores, followed by functional scores and emotional scores [29]. Individuals with CRS and allergic rhinitis have the greatest level of disability [29].

We found that females had higher RSDI and SNOT-22 scores and thus poorer QoL, which is consistent with a Vietnamese study proving CRS females have poorer QoL than men [30]. In our study only 10% of students were active smokers, which is less than the 25% reported in other studies [31]. One explanation for this difference is that the majority of respondents in our study were females, who traditionally do not smoke in our society [31].

The prevalence of chronic rhinosinusitis was higher among medical students compared with nonmedical students: this result is consistent with other study results. This is due to the wide exposure of medical students to the hospital environment. Other factors are nasal polyps and abnormality of the nasal structure [32,33].

A key strength of the present study was the combined use of two different tests, SNOT-22 and RSDI, for the determination of QoL, using the biggest public-sector university enrolment in a real-world setting. Thus, the results mirror actual results and external validity is high. Additionally, this is the first study that we know of that makes a comparison between medical and nonmedical students. Research limitations include the small number of participants, lesser understanding of disease in nonmedical students, cultural aversion to smoking or reluctance to reluctance to share smoking status, absence of previous studies of this type in the region and symptoms being common to other diseases similar to sinusitis

## Conclusion

Medical students were found to suffer more with the symptoms hampering their efficient functioning. Symptoms were found to be aggravated with TRAP exposure. Sinusitis is silently predating the QoL of students, particularly females.

**Summary points**

- Common symptoms of chronic rhinosinusitis (CRS) which affect the quality of life (QoL) are eye pain which makes it difficult to read (28.3%), pain in face which makes it difficult to concentrate (25.3%) and sniffing which irritates friends and family (23.3%).
- Participants' QoL affected based on Sino-nasal Outcome Test showed a mild 45%, moderate 48% and severe 7% effect on QoL. This difference is statistically significant (p = 0.004).
- QoL of CRS male students was affected more compared with female students.
- QoL of CRS health science students was affected more compared with social science students.
